# Stereotactic body radiation therapy and CTLA-4 blockade as a novel therapy for advanced melanoma

**DOI:** 10.1186/2051-1426-1-S1-P118

**Published:** 2013-11-07

**Authors:** Christina Twyman, Andrew Rech, Ramesh Rengan, Robert Vonderheide, Andy Minn

**Affiliations:** 1 Division of Gastroenterology, University of Pennsylvania, Philadelphia, PA, USA; 2 Department of Radiation Oncology, University of Washington School of Medicine, Seattle, WA, USA; 3 Department of Radiation Oncology, University of Pennsylvania, Philadelphia, PA, USA; 4 Division of Hematology Oncology, University of Pennsylvania, Philadelphia, PA, USA; 5 University of Pennsylvania, Philadelphia, PA, USA

## 

Ipilumimab, which is a monoclonal antibody to the inhibitory T-cell receptor CTLA-4, has been shown to improve survival in patients with metastatic melanoma; however, the response rate was only 10%, necessitating the need to develop ways to improve treatment. Anecdotal evidence suggests that stereotactic body radiation (SBRT) might cooperate with anti-CTLA4 to improve response rates both for irradiated and unirradiated tumors, a concept that is being tested in a phase I/II clinical trial as well as a pre-clinical mouse model. Our central hypothesis is that SBRT delivered to an index metastatic lesion will stimulate tumor antigen release and improve the anti-tumor immune response to anti-CTLA4 in advanced melanoma. To pre-clinically model the combination of SBRT and anti-CTLA4, we are using a syngeneic transplantation model with B16-F10 melanoma, which is injected to the bilateral flanks. As expected, response of the primary tumor is generally dictated by whether it is irradiated or not. In the unirradiated secondary tumor, we have observed CD8+ T-cell dependent regression in the SBRT+anti-CTLA4 cohort resulting in improved progression-free survival (77%) compared to anti-CTLA4 alone (33%), SBRT alone (0%), or the control (0%). As not all mice respond to the combined therapy, we have also begun to interrogate potential mechanisms of resistance by trascriptomic profiling of resistant tumors. To test our hypothesis clinically, we have initiated a single-center, investigator-sponsored, phase I/II clinical trial of escalating doses of SBRT with ipilimumab in patients with metastatic melanoma. Patients undergo SBRT to a single metastatic deposit followed by standard-of-care ipilimumab. Two treatment strata are used: bone/lung vs liver/subcutaneous. To date, no dose-limiting toxicities have been observed in the first 12 patients enrolled on this trial. Major regressions of irradiated as well as non-irradiated lesions have been observed. Immune assessments to understand mechanism and resistance are underway. Thus, by understanding the genes and mechanisms associated with the SBRT+anti-CTLA4 response, our goal is to complement an on-going clinical trial with insight into potential biomarkers and therapeutic targets that may improve the selection and treatment of patients with metastatic melanoma.

